# Study on the Structure of Ginseng Glycopeptides with Anti-Inflammatory and Analgesic Activity

**DOI:** 10.3390/molecules23061325

**Published:** 2018-05-31

**Authors:** Haoming Luo, Difu Zhu, Ying Wang, Yinghong Chen, Ruizhi Jiang, Peng Yu, Zhidong Qiu

**Affiliations:** 1 Changchun University of Chinese Medicine, Changchun 130117, China; luo.haoming@163.com; 2 Jilin Jice Inspection Technology Co., Ltd., Changchun 130117, China; zhudifu@163.com (D.Z.); zhongyanjrz@sina.com (R.J.); 3 Jilin Academy of Chinese Medicine and Material Medica Science, Changchun 130012, China; wangying02231@sina.com (Y.W.); zhongyancyh@sina.com (Y.C.)

**Keywords:** *Panax ginseng*, glycopeptides, structure analysis, analgesic effect, inflammatory pain

## Abstract

*Panax ginseng* is well known for its medicinal functions. As a class of important compound of ginseng, ginsenoside is widely studied around the world. In addition, ginseng glycopeptides also showed good biological activity, but researches in this field are rarely reported. In this study, ginseng glycopeptides (Gg) were first prepared from *Panax ginseng* by reflux extracted with 85% ethanol and the following purification with Sephadex G-15 column. Then, the inflammatory pain models induced by carrageenan and the rat pain models induced by Faure Marin were established for research on mechanism of analgesic activities. It is showed that Gg had an obvious inhibiting effect on inflammation and a significant reduction on the Malondialdehyde (MDA) of inflammatory foot tissue. And there were significant differences between moderate to high dose of Gg and model group in Interleukin 1β (IL-1β), Interleukin 2 (IL-2), Interleukin 4 (IL-4), Tumor necrosis factor α (TNF-α) and Histamine. The two models can be preliminarily determined that the analgesic effect of Gg may be peripheral, which mechanism may be related to the dynamic balance between proinflammatory cytokines (TNF-α, IL-1β) and anti-inflammatory cytokines (IL-2, IL-4, and Interleukin 10 (IL-10)). A series of methods were used to study Gg in physical-chemical properties and linking mode of glycoside. The high-resolution mass spectrometry was used for identification of the structure of Gg. Moreover, the structure of 20 major Gg were investigated and identified. The structural analysis of Gg was benefit for the next study on structure-activity relationship.

## 1. Introduction

Ginseng (*Panax ginseng* C.A. Mey) is commonly used as food and as a clinical medicine, not just as a compound or single herbal medicinal decoction. Due to the unique efficacy of drugs, ginseng has been widely considered by many scholars [1,2,3,4]. The composition of ginseng is complex, including ginsenosides, polypeptides, polysaccharide, glycoconjugates components, and other compounds. Among them, ginsenosides are one class of compounds that are extensively studied for their identification and biological activities [5,6,7,8]. Furthermore, there are still other works that have focused on the research of ginseng polypeptides [9]. In recent years, some studies reported that the ginseng polysaccharides and glycoconjugates also have a variety of biological activities [10]. With the improvement of the level of modern research and analysis, more plant glycoconjugates with biological roles will be revealed.

Glycosylation is one of the most significant, and the most widespread post-translational activities of life, which not only affects the biological activity and the conformation of the protein in localization and transport, but also in cell communication, molecular recognition, and signal transduction of specific biological processes [11]. It is concluded that more than 50% of proteins are modified by glycosylation, but most of the glycoproteins have not yet been found due to the high complexity of glycosylation [12,13]. Although some studies have demonstrated the effects of glycosylation on the function of animal proteins, there were few reports about the studies on the structure and the activity of plant glycoprotein until recently. On the other hand, due to its fundamental importance in cell biology, protein glycosylation has also been implicated in a number of human diseases, including congenital muscular dystrophies, cancer [14], alcoholism [15], and Alzheimer’s disease [16]. However, only a few studies focused on plant glycoproteins for the treatment of human diseases.

Pain is a distress feeling caused by tissue damage or potential injury. At present, the treatment of pain is still a major problem in clinical practice, commonly used analgesics are often accompanied by varying degrees of side effects. Nonsteroidal Antiinflammatory Drugs (NSAIDs) can cause gastrointestinal and liver dysfunction, and opioid drugs lead to addiction and tolerance, etc. Ginsenosides are usually considered as effective substances for ginseng’s analgesic activity. Intraperitoneal administration of ginsenosides before or after surgery lead to antihyperalgesic effects. It may be associated with anti-inflammatory cytokines and *N*-methyl-d-aspartate Receptor (NMDA) signaling [17]. Ginsenosides produce antinociceptive effects through their action at the spinal and/or supraspinal site(s), not at nociceptors in the periphery [18]. Ginsenosides Rb1 and Rg have significant anti-inflammatory and analgesic activities, the mechanism is related to antioxidation, inhibition of NO synthesis and inhibition of capsaicin receptor activation [19,20].

In our previous study, glycoproteins from *Panax ginseng* root showed significantly analgesic activity [21], but the mechanism of the effect was not discussed. Therefore, in this paper, the primary mechanism of anti-inflammatory and analgesic effect of ginseng glycopeptides (Gg) was studied based on a carrageenan induced rat inflammatory pain models and a formalin induced rat pain models. In order to clarify the structure of drug effect substances and characterization, the contents of protein, neutral sugar, and uronic acid were determined respectively in Gg. In addition, with the development of the monoclonal antibody drug, the mass spectrometry has been widely used in glycosylation analysis in recent years. So, the structures of Gg were identified by using the Q Exactive mass spectrometry in combination with the methylation analysis here. This work may be beneficial to future research on the relationship between the structure and analgesic efficacy of ginseng glycopeptides.

## 2. Results and Discussion

### 2.1. Preparation of Gg

**Figure 1:**
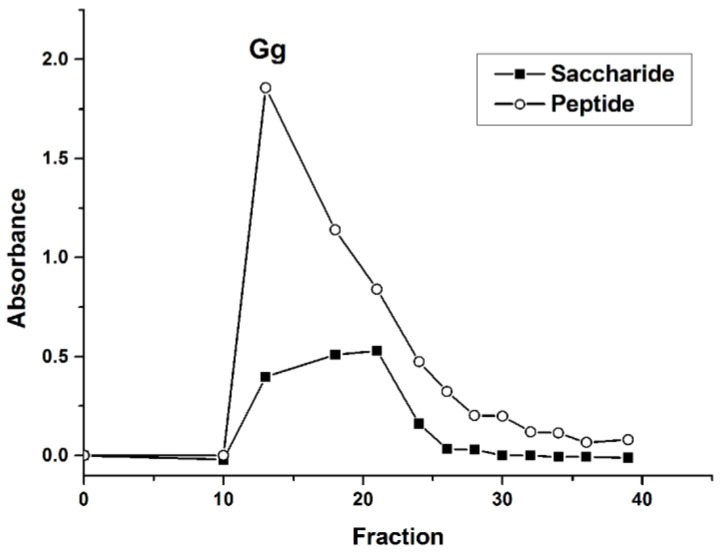
Sephadex G-15 elution profile. Saccharide: absorbance at 490 nm from the phenol-sulfuric acid assay. Peptide: absorbance at 280 nm.

The glycoprotein with analgesic activity were obtained in accordance with our previous works [21]. To further study, glycopeptides were then purified via a Sephadex G-15 column. As shown in Figure 1, the peak of the saccharide coincides with the peak of the peptide. After fraction 25, the peak intensity decreased significantly. Thus, fractions from 10 to 25 were collected, concentrated, and lyophilized, and named Gg.

### 2.2. Inflammatory Pain Induced by Carrageenan

Tissue injury leads to the formation of inflammatory mediators like histamine, 5-hydroxytryptamine, NO and so on. And immune cells further release cytokines and growth factors. Some mediators activate peripheral nocieptors directly and others act indirectly via inflammatory cells to the release of algogenic agents [22]. Cytokines play an important role in inflammatory pain. In addition to their effects on the activation of immune cells, they also have a strong stimulus to the neurons. When the body is stimulated by various kinds of injuries, mononuclear macrophages will release IL-1β, TNF-α and other inflammatory mediators. IL-1β can induce the production of IL-2, IL-6 and other endogenous mediators. IL-1β, IL-2 and TNF-α play a synergistic role in aggravating inflammatory response pain. TNF-α stimulates the production of IL-4 and IL-10 from monocytes, while IL-4 and IL-10 can inhibit the synthesis of IL-1β, IL-6, TNF-αand other mediators, thus forming a cycle of compensatory anti-inflammatory response of their own products [23,24].

**Figure 2:**
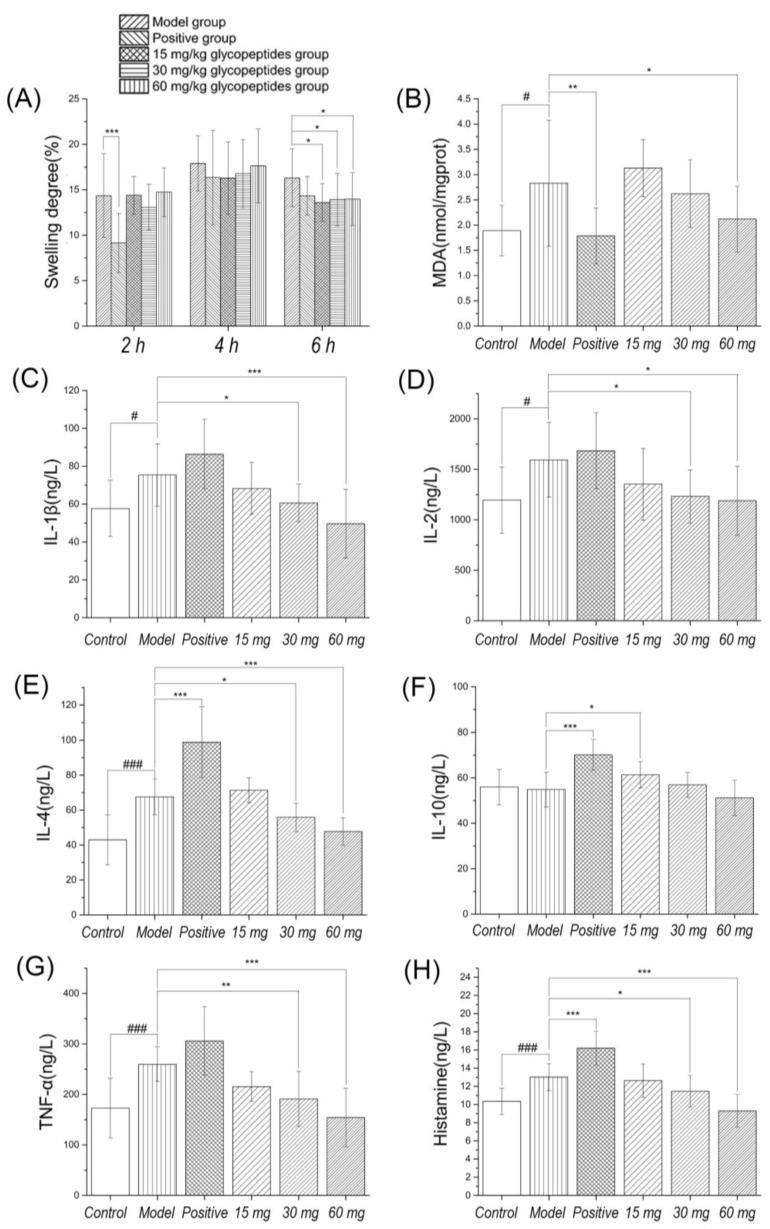
Effect of Gg on foot swelling of rats in the carrageenan inflammatory pain model. (**A**) Effect of Gg on foot swelling degree of rats induced by carrageenan. (**B**) Effect of Gg on MDA of inflammatory foot tissue in the carrageenan inflammatory pain model. (**C**–**H**) Effect of Gg on IL-1β, IL-2, IL-4, IL-10, TNF-α and Histamine of inflammatory foot tissue in the carrageenan inflammatory pain model, respectively. There were significant difference between positive control (Diclofenac sodium) and model group in swelling degree at 2 h, and there were significant differences between low to high dose of Gg and model group in swelling degree at 6 h. There were significant differences between positive control and model group in MDA, IL-4, IL-10 and Histamine. There were significant differences between moderate to high dose of Gg and model group in IL-1β, IL-2, IL-4, TNF-α and Histamine (*p* < 0.05–0.001). Low dose Gg group exhibited only significant increase in IL-10. ^#^ *p* < 0.05, ^###^ *p* < 0.001, * *p* < 0.05, ** *p* < 0.01, *** *p* < 0.001.

After the carrageenan-induced inflammation, the toes of rats showed edema, hyperalgesia and erythema formation, and leads to the release of pro-inflammatory cytokines. Neutrophils transferred to inflammatory sites, producing reactive oxygen species and other inflammatory responses. The time to reach the maximum swelling degree is about 5–6 h [25]. In Figure 2, the results showed that the swelling degree reached the maximum in the 4–6 h group after carrageenan induced inflammation. There was a significant differences in swelling degree between the positive control group and the model group at 2 h, but no significant differences at 4 h and 6 h. The reason may be that diclofenac sodium is a nonsteroidal anti-inflammatory drug which reduces the production of prostaglandins by inhibiting the cyclooxygenase. It has a rapid onset time, and peaked at about 2 h. There were significant differences between low to high dosage of ginseng glycoprotein and the model group at 6 h. It means that the onset time is relatively late, but the action time is longer.

Next, we evaluated the effect of diclofenac sodium and glycopeptides on the levels of pro-inflammatory and anti-inflammatory cytokines. The results showed that diclofenac sodium had the ability to increase the level of IL-4 and IL-10. Although there is no significant difference in TNF-α level, there is an upward trend. The results illustrated that the drug activity decreased at 6 h, the rise of TNF-α could stimulate the production of anti-inflammatory cytokines, and the experimental results were consistent with the increase of IL-4 and IL-10 levels. Glycopeptides could effectively inhibit the level of TNF-α, IL-1β and IL-2 at 6 h, indicating that its analgesic activity may be closely related to its anti-inflammatory ability. Moderate and high dosage group of glycoproteins significantly reduced inflammatory cytokines in rats model induced by carrageenan, and the possible mechanism is inhibition of the synthesis and release of cytokines. Its performance has obvious inhibiting effect on inflammation and MDA in foot tissue was reduced by Gg.

### 2.3. Pain Induced by Faure Marin

**Figure 3:**
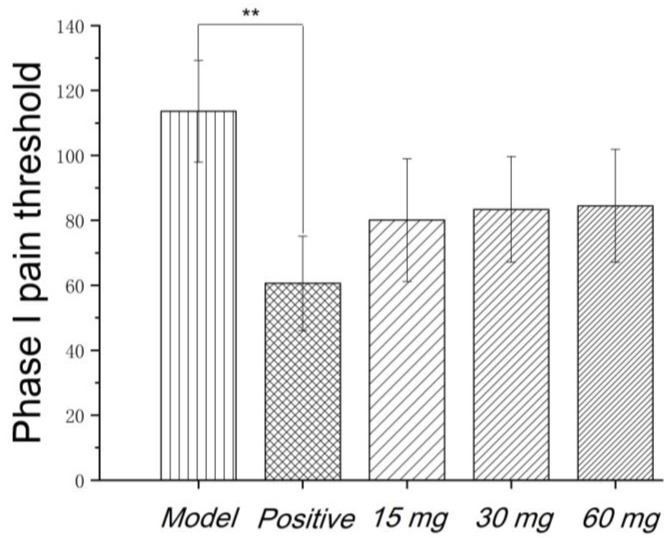
Effect of Gg on phase I pain threshold of rat model induced by Faure Marin. Positive control showed significant inhibitory effects on the pain threshold of phase I. Low to high dose of Gg showed no significant differences with model group. *p* < 0.05–0.01; ** *p* < 0.01.

**Figure 4:**
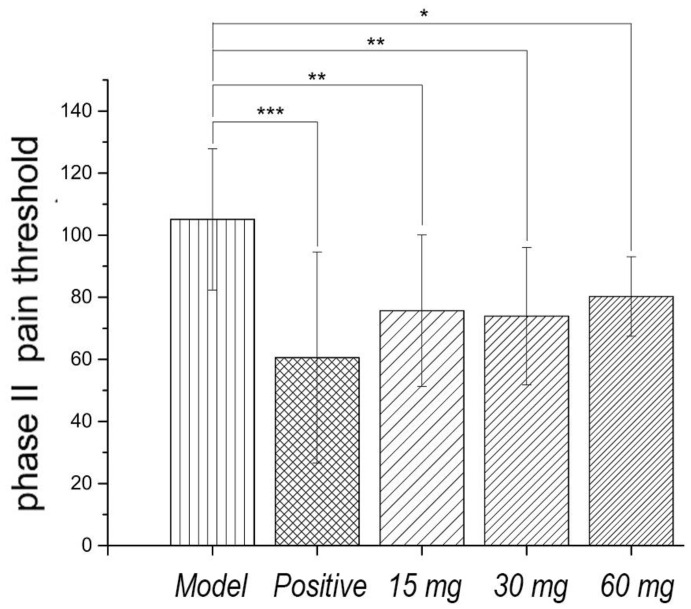
Effect of Gg on phase II pain threshold of rat model induced by Faure Marin. Gg showed significant inhibitory effects on the pain threshold of phase II (*p* < 0.05–0.01); (* *p* < 0.05, ** *p* < 0.01, *** *p* < 0.001).

Faure Marin pain model is a continuous pain model. Pain response is divided into two phases. Phase I (early phase) response continued for 0–5 min, mainly due to stimulation of C fibers. Phase II (late phase) responses continued for 10–60 min when inflammatory mechanism was involved in. Glycoproteins had no significant effects on the pain threshold of phase I. Low to high dosage of glycoproteins significantly inhibited the pain threshold of phase II, indicating that the analgesic effect of glycoproteins may be related to the anti-inflammatory effect, as shown in Figure 3 and Figure 4.

Monoamine neurotransmitters are composed of 5-HT, dopamine, norepinephrine and so on. In this study, all dosage of glycoproteins had no significant effect on 5-HT, DA, NE and other monoamine neurotransmitters in the brain of the pain model rats. It had no significant effect on glutamate and GABA in the brain of the pain model rats, suggesting that the analgesic sites of Gg may not belong to the central nervous system.

The analgesic mechanism of traditional Chinese medicine can be roughly divided into central nervous system and peripheral nervous system. To above two pain models can be preliminarily determined that the analgesic effect of glycoproteins may be a peripheral analgesic effect. The mechanism of its analgesic effect may be related to the dynamic balance between proinflammatory cytokines (TNF-α, IL-1β) and anti-inflammatory cytokines (IL-2, IL-4, and IL-10). The generation of pain is often caused by many factors and the mechanism of analgesic Chinese medicine is quite complex. Further, we will investigate other pathway like endocannabinoid or the kynurenine pathways to clarify the mechanism of the analgesic effect of Gg.

### 2.4. Contents, Molecular Weight, and Monosaccharide Compositions

The total contents of protein, neutral sugar and uronic acid were 85.23%. The results of characterization of Gg were as follows: protein content was 53.91%, neutral sugar content was 33.50%, and uronic acid content was 7.82%. HPLC chromatography showed two peaks, so Gg was a mixture, and the molecular weight of the major ingredient was 4903 Da, calculated by gel permeation chromatography (GPC) software. Monosaccharide compositions were mannose (Man, 21.01%, mass percent content compared with total sugar), glucose (Glc, 26.53%), HexNAc (GalNAc or GlcNAc, 12.17%), galactose (Gal, 28.23%), and fucose (Fuc, 9.78%).

### 2.5. Methylation Analysis

Because of the methylation products of Man, Glc and Gal were the same, which are expressed as Hex. Similarly, GlcNAc and GalNAc are expressed as HexNAc. As we know, the core backbone of *N*-glycan was Man α1→6 (Man α1→3) Man β1→4 GlcNAc β1→4 GlcNAc β1→Asn, called “core pentasaccharide”. Analysis of the results based on methylation. In the high-mannose *N*-linked glycan, the backbone is composed of Man α1→2 Man chain. In the complex *N*-linked glycan, the backbone of biantennary is composed of GlcNAc β1→2 Man chain, and the backbone of triantennary is composed of GlcNAc β1→2 (GlcNAc β1→4) Man chain or GlcNAc β1→2 (GlcNAc β1→6) Man chain. Furthermore, the four-antenna has the same structure as the triantennary. The hybrid *N*-linked glycan contain the mannose branch and the complex branch. In addition, the structure of *O*-glycan is usually simple, whose backbone is made up of the Gal β1→3 GalNAc α1→Ser/Thr. On this basis, glycosyltransferases add sugar residues to the core glycan structure, giving rise to the three main types of glycans: high-mannose, complex and hybrid glycans [12]. The methylation analysis has proved the existence of the *N*- or *O*-linked glycan above in the Gg sample as shown in Table 1. These data would be used for the analysis of glycopeptide structure next.

### 2.6. Analysis of Glycopeptide Structure

**Figure 5:**
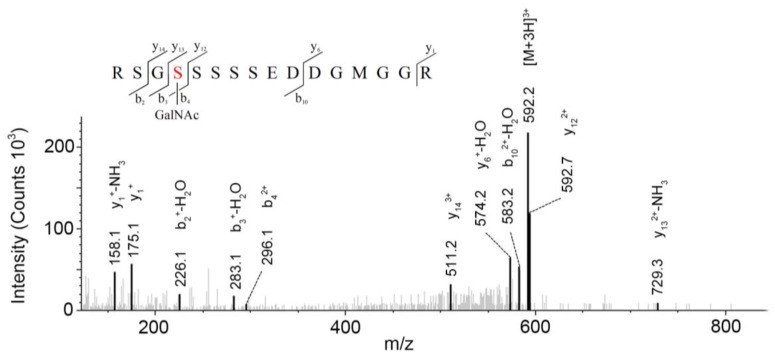
MS/MS spectra of glycopeptides whose peptide backbone is RSGSSSSSEDDGMGGR. The spectra are annotated according to the correct assignment which is RSGS(GalNAc)SSSSEDDGMGGR.

LC-MS/MS-based approaches were utilized to achieve a deep site-specific structural characterization of the Gg sample. The spectrum was searched for peaks potentially representing glycopeptides. And the masses of all possible glycopeptides were calculated by adding the masses of peptide and glycan [26]. To further investigate the ginseng glycopeptides, the raw LC-MS/MS data were searched in the Uniprot ginseng protein sequence database by using Byonic software to determine the structures. For example, as shown in Figure 5, glycopeptides were observed in the MS/MS spectra. This spectrum contains peaks of charge states from 1+ to 3+. The y ion series along with b fragments confidently identify the modified sequence, and the site of modification can be restricted. A strong signal of the [M + 3H]^3+^ ion at *m*/*z* = 592.2 for the respective triply-charged glycopeptide was present, so the molecular weight was 1773.7 Da. In Figure 4, the ion y_12_^2+^ was at *m*/*z* = 592.73566 and the ion y_13_^3+^ − NH_3_ was at *m*/*z* 729.28955, which data revealed the glycosylation site was at S4 and the linked monosaccharide was HexNAc. Furthermore, some studies indicated that *O*-Glycans were α-linked to the hydroxyl group of serine or threonine residues via GalNAc [9]. Accordingly, we determined that the structure of this glycopeptide was RSGS(GalNAc)SSSSEDDGMGGR.

**Figure 6:**
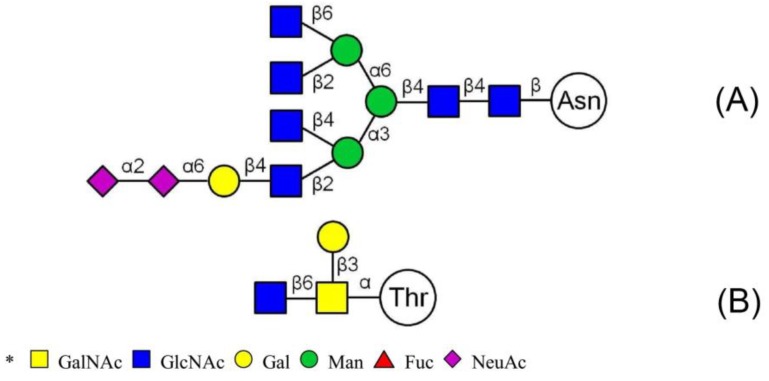
Structures of two glycans linked to the peptide NTSYTAVR. (**A**) *N*-glycan: HexNAc(6)Hex(4)NeuAc(2) and (**B**) *O*-glycan: HexNAc(2)Hex(1).

LC-MS/MS analysis provides an effective method to determine the glycosylation sites of glycan. Meanwhile, preliminary analysis of the structures of *N*-glycan and *O*-glycan are conducted according to results of methylation analysis and knowledge of the literature. Thus, combined with these two strategies, many more complex structures of glycopeptides could be identified. For example, a glycopeptides whose peptide backbone is “NTSYTAVR” has been analyzed by LC-MS/MS, which containing two sugar chains: the *N*-glycan is HexNAc(6)Hex(4)NeuAc(2) linked to Asn(N1) and the *O*-glycan is HexNAc(2)Hex(1) linked to Thr(T2). Some research indicated that the “core pentasaccharide” structure of *N*-glycan is essentially made up of two GlcNAc and three Man residues [27]. This core glycan was then elaborated and modified further, resulting in a diverse range of *N*-glycan structures. Thus, the structure of HexNAc(6)Hex(4)NeuAc(2) was considered as a “four-antenna” structure of the complex *N*-glycan, as shown in Figure 6A. It is should be noted that the glycosidic bond between two terminal sialic acids was α2-8-linked in order to form lactone for stability [28]. In addition, *O*-glycan is usually the addition of GalNAc to Ser or Thr residues by *N*-acetylgalactosaminyltransferase, followed by other carbohydrates, such as Gal, as shown in Figure 6B. These results are consistent with the results of the methylation analysis above. Consequently, the structure of glycopeptide “NTSYTAVR” has been determined by this strategy.

Based on analysis of the Byonic software, the glycosylation sites and the glycan composition of glycopeptides were determined. Then, the structures of each glycan were deduced preliminarily with the help of methylation analysis data. By this combination strategy, several major glycopeptides were identified from Gg, which included both *N*-linked and *O*-linked glycopeptides, as shown in Table 2. In *N*-glycopeptides, there are three main types of glycans: complex, high mannose and hybrid glycans. But in *O*-glycopeptides, the structures of glycan chains are more diverse and shorter. Additionally, in some glycopeptides, it is found that there are both *N*-glycan and *O*-glycan linked to the appropriate locations. Meanwhile, these peptides were described by searching against the Uniprot ginseng protein sequence database, as shown in supporting information Table S1. In botany, the present studies were limited to the effects of plant peptides on their growth or physiological functions. Dammarenediol-II synthase, for instance, is the first enzyme in the biosynthesis of ginsenosides. Most ginsenosides are composed of dammarenediol-II aglycone with various sugar moieties [29]. However, our group focused on the study of plant glycopeptides for the treatment of human diseases. On the basis of this research, the study of relationship between structure and activity will be the key work in the future.

## 3. Materials and Methods

### 3.1. Preparation of Gg

The roots of *Panax ginseng* were obtained from a local market (Changchun, China), and authenticated by Prof. Jiang (Jilin Academy of Chinese Medicine and Material Medical Science, Changchun, China). The roots (1 kg) were first pulverized by grinder and reflux extracted with 85% ethanol to remove the saponine. The residue was decocted with 5 L water three times, and then the rinsing fluids were merged into one portion, and evaporated to 200 mL. Subsequently, the aqueous extract was forced through a cellulose superfiltration system (molecular weight cut-off is 20,000 Da) to give two different molecular weight fractions. The low molecular weight fraction was dialyzed against water by using a dialysis sac with a 1000 Da cut-off. The contents out of the dialysis sac were collected, concentrated, lyophilized and loaded on a Sephadex G-15 column (Ф2.5 × 95 cm, from Sigma Chemical Co., Saint Louis, MO, USA), which eluted with pH 7.0 phosphate-buffered saline at a flow rate of 0.2 mL/min, to obtain purified glycopeptide. The eluent was collected by a fraction collector, and named Gg, and then purification of the glycopeptides was carried out by HPLC. The two major portions were collected for further characterizing.

### 3.2. Inflammatory Pain Induced by Carrageenan

60 male SD rats were randomly divided into 6 groups: control group, model group, positive group (diclofenac sodium, 9.5 mg/kg), low dosage group (15 mg/kg), moderate dosage group (30 mg/kg) and high dosage group (60 mg/kg). The positive group was treated by intragastric administration of diclofenac sodium and the model group was intraperitoneally injected with equal volume of normal saline once a day for 4 days. The glycoproteins groups were injected into the abdominal cavity of rats in each group according to the above dose. Except the normal control group, rats were given drugs for the fourth day, at the same time, the right toes were injected by intradermal of 1% carrageenan 0.1 mL after 5 h modeling for the reagent.

Outcomes: The circumferences of feet were recorded at 2 h, 4 h and 6 h after modeling, and the swelling degrees at each time point were calculated.

Swelling degree = (the circumference of right foot − the circumference of left foot)/the circumference of left foot × 100%

The test results: after modeling 6h, the rats were treated with 3% chloral hydrate 1 mL/100 g anesthesia, abdominal aorta blood collection, which was centrifuged at 3000 rpm for 10 min, serum was collected according to the ELISA kit manual test index: IL-1β, IL-4, IL-2, IL-10, TNF-α, histamine.

0.3 g inflammatory foot tissue and normal saline were made into 10% homogenate. Centrifuge the homogenate at 3000 rpm for 10 min at low temperature, and MDA was measured by the operation method of the supernatant kit.

### 3.3. Pain Induced by Faure Marin

65 male SD rats were randomly divided into 6 groups: control group, model group, positive group (diclofenac sodium, 9.5 mg/kg), low dosage group (15 mg/kg), moderate dosage group (30 mg/kg) and high dosage group (60 mg/kg). There were 10 rats in the control group and 11 rats in the other groups. The positive group was intraperitoneally injected with tramadol hydrochloride 5 mg/kg, and the model group was injected with equal volume of normal saline intraperitoneally, once a day for 5 days. The glycoprotein group was intraperitoneally injected in each group according to the above dose. At the time of 30 min after the last time point with drugs given, except the normal control group, the other rats left after subcutaneous were injected by 5% Formaldehyde Solution 0.05 mL, which caused pain.

Outcomes: After injection of formaldehyde, the rats were immediately placed in a 2000 mL beaker. The beaker was placed on a glass platform, and a 30 degree mirror was placed under the glass table. Pain reactions of the rats were observed from the mirror surface. The pain response status and pain grading of rats were recorded from 1 to 5 min (phase I (early phase)), 31 min and 35 min (phase II (late phase)) after modeling.

Classification of pain: 3 points = lick, bite, or shake; 2 points = raise the foot; 1 point = touch the bottom but not weight, limp when walking; 0 point = normal weight bearing. The number of seconds for each minute of the reaction was recorded multiplied by the corresponding response score, and the sum of the product was a quantitative index of pain.

At the end of the observation, the rats were killed after 30 min, 0.3 g of brain tissue was taken, and the homogenate of normal saline was added to make 10% homogenate liquid. The homogenate was centrifuged at 3000 rpm for 10 min at low temperature to take the supernatant. According to the instructions of the ELISA kit, 5-serotonin (5-HT), norepinephrine (NE), dopamine (DA), glutamic acid (Glu), and gamma aminobutyric acid (GABA) were detected.

### 3.4. Determination of Characteristics

The contents of protein, neutral sugar, and uronic acid were determined by the Bradford methods [30], phenol-sulfuric acid [31], and carbazole [32] using bovine serum albumin (≥99%, Sigma Chemical Co.), d-glucose (≥99%, National institutes for food and drug control, Beijing, China), d-glucuronic acid (≥99%, Sigma Chemical Co.) and as the respective standards.

HPLC (Shimadzu, Kyoto, Japan) equipped with a refractive index detector (Shimadzu, Kyoto, Japan) on a SRT SEC-100 column (Sepax Technologies, Newark, DE, USA) and with GPC software (Beijing Longzhida Software Co., Ltd., Beijing, China) were used to obtain the purity and average molecular mass. l-tryptophan (≥99%, Beijing Dingguo Biotechnology Co., Ltd., Beijing, China), vitamin B12 (≥99%, Gen-view Scientific Inc., Arcade, NY, USA), aprotinin (≥98%, Amresco LLC., Solon, OH, USA), cytochrome C (≥95%, Sigma Chemical Co.), and bovine serum albumin (≥99%, Sigma Chemical Co.) were used as standards.

The monosaccharide compositions were analyzed as 3-Methyl-1-phenyl-2-pyrazoline-5-one (PMP) derivatization using HPLC with a UV detector on a C18 column [33].

### 3.5. Methylation Analysis

The samples of Gg were methylated once by the Ciucanu method [34]. The resulting partially-methylated alditol acetates were analyzed by gas chromatography-mass spectrometer (GC-MS). GC-MS was performed on a Trace-MS (Fingan, San Francisco, CA, USA) using a DB-17HT capillary column (30 m × 0.25 mm, J and W Scientific, Folsom, CA, USA). The injection temperature was 200 °C. The column temperature was kept at 50 °C for 2 min after sample injection, increased to 150 °C at 50 °C/min, kept at 150 °C for 1 min, then increased to 250 °C at 4 °C/min. The mass spectra were recorded in the positive ion electron ionization mode.

### 3.6. LC-MS/MS Analysis

#### 3.6.1. Sample Preparation

Disulfide bonds of the samples were reduced for 40 min with 5 mM dithiothreitol at room temperature and alkylated for 40 min with 15 mM iodoacetamide in the dark. The mixtures were acidified with trifluoroacetic acid (TFA) to 1%, and desalted by a home-made C18 tip. Finally, the desalted samples were dried in a vacuum concentrator and purified samples were produced for analysis by nanoLC-MS/MS. The dried samples were dissolved in 10 μL of 0.1% formic acid in water and subjected to nanoLC-MS/MS analysis.

#### 3.6.2. Assay of High-Resolution Mass Spectrometry

The samples were analyzed by LC-MS using a nanoflow RP-HPLC online-coupled to a Q Exactive (Thermo Fisher Scientific, Bremen, Germany) mass spectrometer operating in the positive ion mode. The nano-flow was eluted at a flow rate of 300 nL/min [35] with solvent A (0.1% formic acid in water) and solvent B 0.1% formic acid in acetonitrile). LC analysis was performed by a 61 min staged gradient elution program, 0–2 min (5% B), 2–50 min (5–30% B), 50–52 min (30–100% B), and 52–61 min (100% B).

The microelectrospray ion source was operated at 2.2 kV. Full-scan MS spectra were acquired from *m*/*z* 300–30,000 Dalton. Data collection used Orbitrap for the full-scan MS, and then proceeded to isolate the top five ions for MS/MS by high energy collision dissociation (HCD).

#### 3.6.3. Identification of the Key Peptides

The raw MS files were analyzed and searched against the Uniprot ginseng protein sequence database using Byonic software (Version 2.3.5, Protein Metrics Inc., San Francisco, CA, USA). The parameters were set as follows: the protein modifications were carbamidomethylation (C) (fixed), oxidation (M) (variable), and glycosylation (*O*-linked and *N*-linked); the enzyme specificity was set to trypsin; the maximum missed cleavages was set to 2; the precursor ion mass tolerance was set to 10 ppm; and MS/MS tolerance was 0.6 Da. The cutoff of FDR for peptide identification was set to <1%.

### 3.7. Statistical Analysis

Each indexes were expressed in ±s, and the statistical software SPSS (17.0, International Business Machines Corporation, Armonk, NY, USA) was used to carry out a single factor analysis of variance (One-way ANOVA) between groups, with a significant difference of *p* < 0.05. *P* value refers to the probability that a statistical summary (such as the mean difference of two groups of samples) is the same or even larger as the actual observation data in a probability model.

## 4. Conclusions

In summary, a group of ginseng glycopeptide samples obtained by dialysis and Sephadex G-15 column purification was investigated. The carrageenan induced rat inflammatory pain models and formalin induced rat pain models were established for the study on mechanisms of analgesia. In these two models, Gg showed long aging analgesic effect. The experimental results indicated that Gg may possess the effect of peripheral analgesic. At present, ginsenosides are considered to be the active substance of ginseng. The research about its anti-inflammatory and analgesic activities mainly focused on ginsenosides, Rb1 and Rg1. We paid close attention to glycopeptides which are active macromolecules, and this is a new exploration for the anti-inflammatory and analgesic substances of ginseng. Meanwhile, We have used the method of LC-MS/MS to characterize its structure, this pattern not only rest on the analysis of amino acids and the structure of glycosides, but concern about the connection and bind sites of some proteins and glycosides. According to the data of the methylation analysis, the structures of *N*-glycan and *O*-glycan linked to the peptide chains were subsequently determined. This study laid the foundation for further research on plant glycopeptides for the treatment of human diseases.

## Figures and Tables

**Table 1:** Results of the methylation analysis.

Retention Time (min)	Methylation Product	Structure Reasoning *	
9.587	2,3,4-Me_3_-Fuc	terminal	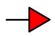
10.800	2,3,4,6-Me_4_ Hex	terminal	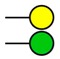
11.080	3,4,6-Me_3_ HexNAc	terminal	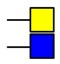
11.940	3,4,6-Me_3_ Hex	mannose chain of *N*-glycan	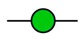
11.997	2,3,4-Me_3_ Hex	chain of *N*-glycan	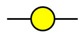
12.000	2,4,6-Me_3_ Hex	chain of *O*-glycan	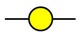
12.210	3,4-Me_2_ Hex	triantennary branch of *N*-glycan	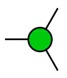
12.293	3,6-Me_2_ Hex	four-antenna branch of *N*-glycan	
12.420	2,4-Me_2_ Hex	core backbone branch of *N*-glycan	
12.973	4,6-Me_2_ HexNAc	chain of *O*-glycan	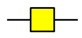
13.343	3,6-Me_2_ HexNAc	chain of *N*-glycan	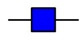
14.257	4-Me_1_ HexNAc	chain of *O*-glycan	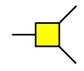
14.263	3-Me_1_ HexNAc	chain of *N*-glycan	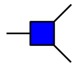

* 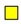 GalNAc 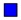 GlcNAc 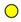 Gal 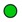 Man 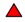 Fuc 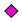 NeuAc.

**Table 2:** Structures of the major glycopeptides from Gg.

Linkage	Structures of Glycopeptides *	
*N*-glycan	complex	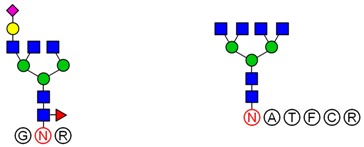
	oligomannose	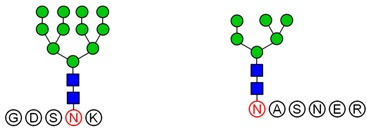
	hybrid	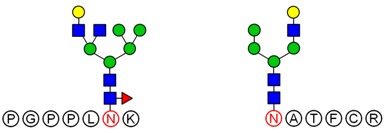
*O*-glycan	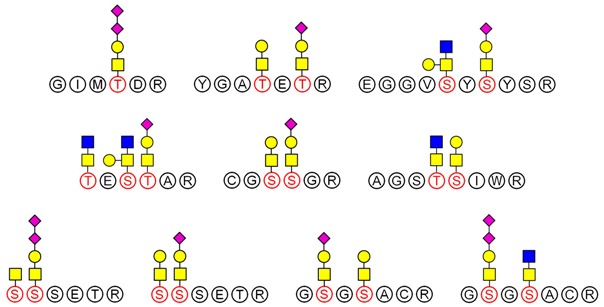	
*N*-glycan & *O*-glycan	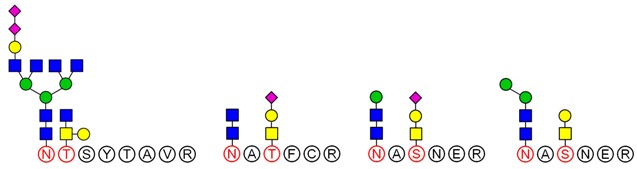	

* 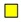 GalNAc 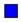 GlcNAc 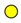 Gal 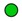 Man 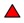 Fuc 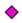 NeuAc.

## Supplementary Materials

Information of major ginseng glycopeptides is available in Support information.
