# Supplementary material for: Study on the Structure of Ginseng Glycopeptides with Anti-Inflammatory and Analgesic Activity
Source: Molecules. 2018 May 31;23(6):1325. doi: 10.3390/molecules23061325 (PMC6099564; doi:10.3390/molecules23061325)
Supplement: Supplementary file 1 [file molecules-23-01325-s001.pdf]

Study on the structure of ginseng glycopeptides with anti-inflammatory and analgesic activity

Haoming Luo [1], Difu Zhu [2], Ying Wang [3], Yinghong Chen [3], Ruizhi Jiang [2, 3], Peng Yu [1, *] and Zhidong Qiu [1, *]

[1] Changchun University of Chinese Medicine, Changchun 130117, China; luo.haoming@163.com

[2] Jilin Jice Inspection Technology Co., Ltd., Changchun 130117, China

[3] Jilin Academy of Chinese Medicine and Material Medica Science, Changchun 130012, China

* Correspondence: cczyyupeng@163.com and qzdcczy@163.com

Table S1. Information of major ginseng glycopeptides from Uniprot Ginseng protein sequence database by using Byonic software.

No.	Peptides Sequence	Glycosylation Modifications	Theo. MH+ [Da]	Byonic Score	Description [Panax ginseng]
1	TESTAR	HexNAc(1)Hex(1)NeuAc(1) [T4]; HexNAc(2) [T1]; HexNAc(2)Hex(1) [S3]	2294.924	34.24	ribosomal protein S18 (chloroplast)
2	GIMTDR	HexNAc(1)Hex(1)NeuAc(2) [T4]	1639.663	42.73	ribosomal protein S8 (chloroplast)
3	EGGVSYSYSR	HexNAc(1)Hex(1)NeuAc(2) [S7]; HexNAc(2)Hex(1) [S5]	2620.03	47.98	ribosomal protein L20 (chloroplast)
4	CGSSGR	HexNAc(1)Hex(1) [S3]; HexNAc(1)Hex(1)NeuAc(2) [S4]	1935.711	3.98	WRKY2 transcription factor
5	AGSTSIWR	HexNAc(1)Hex(1) [S5]; HexNAc(2) [T4]	1648.743	70.37	pleiotropic drug resistance transporter 1
6	YGATETR	HexNAc(1)Hex(1) [T4]; HexNAc(1)Hex(1)NeuAc(1) [T6]	1818.738	72.81	dammarenediol-II synthase
7	GNR	HexNAc(5)Hex(4)Fuc(1)NeuAc(1) [N2]	2446.945	29.97	dammarenediol II synthase
8	SSSETR	HexNAc(1) [S1]; HexNAc(1)Hex(1)NeuAc(2) [S2]	1816.707	19.98	cytochrome c biogenesis FC (mitochondrion)
9	SSSETR	HexNAc(1)Hex(1) [S1]; HexNAc(1)Hex(1)NeuAc(1) [S2]	1687.665	30.67	cytochrome c biogenesis FC (mitochondrion)
10	GSGSACR	HexNAc(1)Hex(1) [S4]; HexNAc(1)Hex(1)NeuAc(1) [S2]	1715.653	33.07	mevalonate diphosphate decarboxylase
11	GSGSACR	HexNAc(1)Hex(1)NeuAc(2) [S2]; HexNAc(2) [S4]	2047.775	17.73	mevalonate diphosphate decarboxylase
12	NATFCR	HexNAc(1)Hex(1)NeuAc(1) [T3]; HexNAc(2) [N1]	1830.732	90.58	UDP-glycosyltransferase
13	NATFCR	HexNAc(3)Hex(5) [N1]	2187.847	26.98	UDP-glycosyltransferase
14	NATFCR	HexNAc(6)Hex(3) [N1]	2472.98	100.73	UDP-glycosyltransferase
15	NASNER	HexNAc(2)Hex(6) [N1]	2068.792	110.26	Cytokinin-beta-glucosidase
16	NASNER	HexNAc(1)Hex(1) [S3]; HexNAc(2)Hex(2) [N1]	1785.713	4.92	Cytokinin-beta-glucosidase
17	NASNER	HexNAc(1)Hex(1)NeuAc(1) [S3]; HexNAc(2)Hex(1) [N1]	1914.755	10.78	Cytokinin-beta-glucosidase
18	NTSYTAVR	HexNAc(2)Hex(1) [T2]; HexNAc(6)Hex(4)NeuAc(2) [N1]	3928.548	29.3	pleiotropic drug resistance transporter 1
19	GDSNK	HexNAc(2)Hex(11) [N4]	2708.976	29.97	Cytochrome P450 CYP71D313
20	PGPPLNK	HexNAc(4)Hex(6)Fuc(1) [N6]	2653.112	29.97	Cytochrome P450 CYP71D313

Table S2. High score glycoproteins (not listed in the main text) Top 20

	Peptide	Glycans	Protein Name
1	R.T[+656.228]MGDR.L	HexNAc(1)Hex(1)NeuAc(1)	PANGI 3-hydroxy-3-methylglutaryl coenzyme A reductase
2	R.T[+802.286]MGDR.L	HexNAc(1)Hex(1)Fuc(1)NeuAc(1)	PANGI 3-hydroxy-3-methylglutaryl coenzyme A reductase
3	R.T[+568.212]MGDK.L	HexNAc(2)Hex(1)	PANGI 3-hydroxy-3-methylglutaryl coenzyme A reductase
4	K.S[+673.243]MDR.S	HexNAc(1)Hex(2)Fuc(1)	PANGI 3-hydroxy-3-methylglutaryl coenzyme A reductase
5	R.FST[+365.132]VKR.A	HexNAc(1)Hex(1)	PANGI 3-hydroxy-3-methylglutaryl coenzyme A reductase
6	K.S[+673.243]MDR.S	HexNAc(1)Hex(2)Fuc(1)	PANGI 3-hydroxy-3-methylglutaryl coenzyme A reductase
7	R.S[+656.228]TEM.-	HexNAc(1)Hex(1)NeuAc(1)	PANGI 3-hydroxy-3-methylglutaryl coenzyme A reductase
8	R.VS[+568.212]HLRFR.G	HexNAc(2)Hex(1)	PANGI 40S ribosomal protein S4
9	K.ILWRT[+974.370]IR.G	HexNAc(4)Hex(1)	PANGI 60S ribosomal protein L13a (Fragment)
10	K.T[+511.190]FFWR.N	HexNAc(1)Hex(1)Fuc(1)	PANGI 60S ribosomal protein L27
11	K.S[+568.212]M[+15.995]R.D	HexNAc(2)Hex(1)	PANGI Actin 1
12	K.S[+947.323]M[+15.995]R.D	HexNAc(1)Hex(1)NeuAc(2)	PANGI Actin 1
13	K.S[+1238.418]M[+15.995]R.D	HexNAc(1)Hex(1)NeuAc(3)	PANGI Actin 1
14	K.S[+892.317]M[+15.995]R.D	HexNAc(2)Hex(3)	PANGI Actin 1
15	K.ILT[+860.327]ER.G	HexNAc(2)Hex(1)Fuc(2)	PANGI Actin 1
16	R.LS[+406.159]HLGLK.D	HexNAc(2)	PANGI ADP-ribosylation factor-like protein
17	K.PS[+406.159]PTR.S	HexNAc(2)	PANGI Aluminum-induced protein
18	K.S[+673.243]MHR.H	HexNAc(1)Hex(2)Fuc(1)	PANGI Beta-Amyrin Synthase 2
19	R.EGGEAS[+365.132]DK.P	HexNAc(1)Hex(1)	PANGI Calcium-dependent protein kinase 1
20	R.EGGEAS[+203.079]DK.P	HexNAc(1)	PANGI Calcium-dependent protein kinase 1

Table S3. Information of fragments from glycopeptides “RSGS(GalNAc)SSSSEDDGMGGR”.

#1	b^{+}	b^{2+}	b^{3+}	Seq.	y^{+}	y^{2+}	y^{3+}	#2
1	157.10839	79.05783	53.04098	R				16
2	244.14042	122.57385	82.05166	S	1618.61331	809.81030	540.20929	15
3	301.16188	151.08458	101.05881	G	1531.58129	766.29428	511.19861	14
4	591.27328	296.14028	197.76261	S-HexNAc	1474.55982	737.78355	492.19146	13
5	678.30531	339.65629	226.77329	S	1184.44842	592.72785	395.48766	12
6	765.33734	383.17231	255.78396	S	1097.41639	549.21183	366.47698	11
7	852.36937	426.68832	284.79464	S	1010.38436	505.69582	337.46631	10
8	939.40139	470.20434	313.80532	S	923.35234	462.17981	308.45563	9
9	1068.44399	534.72563	356.81951	E	836.32031	418.66379	279.44495	8
10	1183.47093	592.23910	395.16183	D	707.27771	354.14250	236.43076	7
11	1298.49787	649.75257	433.50414	D	592.25077	296.62902	198.08844	6
12	1355.51934	678.26331	452.51130	G	477.22383	239.11555	159.74613	5
13	1486.55982	743.78355	496.19146	M	420.20236	210.60482	140.73897	4

14	1543.58129	772.29428	515.19861	G	289.16188	145.08458	97.05881	3
15	1600.60275	800.80501	534.20577	G	232.14042	116.57385	78.05166	2
16				R	175.11895	88.06311	59.04450	1
